# Supplementary material for: Clinical Characteristics and Disease Course of Fibrosing Interstitial Lung Disease Patients in a Real-World Setting
Source: Medicina (Kaunas). 2023 Jan 31;59(2):281. doi: 10.3390/medicina59020281 (PMC9961403; doi:10.3390/medicina59020281)
Supplement: Supplementary file 1 [file medicina-59-00281-s001.zip › medicina-2127495-supplementary.pdf]

# Supplementary Material

## ILD diagnoses

| ILD diagnoses | ICD |
|---|---|
| NSIP (non-specific interstitial pneumonia) | J84.0, J84.1, J84.8, J84.9 |
| Unclassifiable IIP (idiopathic interstitial pneumonia) | J84.0, J84.1, J84.8, J84.9 |
| HP (hypersensitivity pneumonitis) | J67.* |
| Exposure-related ILD | J60, J61, J62, J63.*, J64 |
| Sarcoidosis | D86.0, D86.2 |
| RA-ILD (rheumatoid arthritis associated ILD) | J99.0*M05.1 / M05.1*J99.0, M45, M05.0, M05.1, M05.8, M05.9, M06.* + J84.1, J84.8, J84.9 |
| SSc-ILD (systemic sclerosis-associated ILD) | M34.0, M34.1, M34.8, M34.9 + J84.1, J84.8, J84.9 |
| Mixed CTD (connective tissue disease) | M35.1 |
| Other diffuse CTD | M32.* + J84.*, M33.0 + J84.*, M33.1 + J84.*, M33.9 + J84.*, M33.2 + J84.*, M35.0 |
| IPF | J84.0, J84.1, J84.8, J84.9 |

## Comorbidities

Diabetes

| | |
|---|---|
| E10.x (x = 1,2,5,6,7,9) | juvenile diabetes |
| E11.x (x = 1,2,3,5,6,7,8,9) | adult diabetes |
| E14.9 | undefined diabetes |

Obesity

| | |
|---|---|
| E66.x (x = 0,2,8,9) | obesity |

Sleep apnea

| | |
|---|---|
| G47.3 | sleep apnea |

Cardiovascular diseases

| | |
|---|---|
| I11.x | hypertension |
| I12.x | renal insufficiency related to hypertension |
| I13.x | hypertensive heart and chronic kidney disease |
| I15.x | hypertension related renal insufficiency |

| | |
|---|---|
| I20.x | angina pectoris |
| I21.x | myocardial infarction |
| I22.x | myocardial infarction |
| I24.x | ischemic heart disease |
| I25.x | ischemia |
| I63.x | stroke |
| G46.x | cerebral pulsy |

Renal insufficiency

| | |
|---|---|
| N17.x | renal insufficiency |
| N18.x | renal insufficiency |

Chronic lower respiratory diseases

| | |
|---|---|
| J43.9 | lung emphysema |
| J44.x | COPD |
| J45.x | asthma |
| J46 | acute severe asthma |

Malignant diseases

| | |
|---|---|
| C00-C97 (Cx) | malignant diseases (cancer) |

Mood and other anxiety disorders

| | |
|---|---|
| F30. | maniac disorder |
| F31. | bipolar disorder |
| F32., F33., | depression |
| F34., F38. | mood disorders |
| F41.x | other anxiety disorders |

**ATC codes of medications**

Immunosuppressants

| | |
|---|---|
| azathioprine | L04AX01 |
| methotrexate | L04AX03 |
| | L01BA01 |

TNF-alpha inhibitors

| | |
|---|---|
| etanercept | L04AB01 |
| infliximab | L04AB02 |
| adalimumab | L04AB04 |
| sertolimumab | L04AB05 |

| | |
|---|---|
| golimumab | L04AB06 |
| selective immunosuppressants | |
| abatasept | L04AA24 |
| apremilast | L04AA32 |
| leflunomid | L04AA13 |
| JAK inhibitors | L04AA29 |
| | L04AA37 |
| | L04AA44 |
| mycophenolate | L04AA06 |
| IL-6 inhibitors | |
| tosilitzumab | L04AC07 |
| IL-1 -blocker | |
| anakinra | L04AC03 |
| aurothiomalate | M01CB01 |
| chlorocine | |
| huydroxychlorocine | P01BA01 |
| B cell inhibitor | |
| rituximab | L01XC02 |
| glucocorticoids | |
| dexamethasone | H02AB02 |
| methylprednisolon | H02AB04 |
| prednisolon | H02AB06 |
| sulfasalazine | A07E01 |

**Codes for acute hospitalizations**

A40.3, A40.8, A41.0, A41.1, A41.5, A41.8, A41.9, A49.9, B34.0, B59+, D86.0, D86.2, D86.8, D86.8+I41.8, D86.8+N16.2, D86.9, J10.0, J11.0, J12.1, J12.8, J13, J15.9, J18.9, J20.9, J21.0, J22, J32.0, J32.9, J47, J61, J62.8, J63.3, J67.9, J69.0, J69.8, J80, J82, J84.1, J84.8, J84.9, J92.0, J92.9, J93.1, J93.9, J94.8, J96.0, J96.1, J98.0, R04.2, R04.9, R05, R06.0, R06.2

## Regular expressions used to extract 6MWT and lowest SpO2

```
library(RtoolsKTP)
library(stringr)

## 6 MIN WALKING TEST ##

sentence_data <- extract_sentences_from_text(text_data$TEXT, horizontal = F)

sentence_data_sub <- sentence_data[grepl("6 min|teoreettisesta etä", sentences)]

# % FROM THEORETICAL DISTANCE

sentence_data_sub[, REL_DIST := str_extract(str_extract(sentences, "(\\d+[.,]?\\d*)\\s?\\%\\s?"),
"\\d+[.,]?\\d*")]

# LOWEST SpO2

sentence_data_sub_spo2 <- sentence_data[grepl("alim", sentences)]

sentence_data_sub_spo2[, MIN_SPO2 := str_extract(str_extract(sentences,
"(?<=alimmillaan|alimillaan|alimilleen|alimmilleen).*(\\d+[.,]?\\d*)\\s?\\%\\s?"), "\\d+[.,]?\\d*")]
```

## Fibrosis status during the follow-up years 1-5

| Patient Group | 1 IPF (N=110) | 1 PPF (N=142) | 1 F-ILD (N=194) | 1 F-ILD-U (N=138) | 2 IPF (N=101) | 2 PPF (N=142) | 2 F-ILD (N=192) | 2 F-ILD-U (N=89) | 3 IPF (N=79) | 3 PPF (N=130) | 3 F-ILD (N=186) | 3 F-ILD-U (N=59) |
|---|---|---|---|---|---|---|---|---|---|---|---|---|
| **Fibrosis status** | | | | | | | | | | | | |
| Increased | 24 (21.8 %) | 10 (7.0 %) | 3 (1.5 %) | 2 (1.4 %) | 18 (17.8 %) | 11 (7.7 %) | 3 (1.6 %) | 3 (3.4 %) | 15 (19.0 %) | 3 (2.3 %) | 5 (2.7 %) | 1 (1.7 %) |
| Decreased | 0 (0.0 %) | 0 (0.0 %) | 2 (1.0 %) | 0 (0.0 %) | 0 (0.0 %) | 0 (0.0 %) | 3 (1.6 %) | 0 (0.0 %) | 0 (0.0 %) | 0 (0.0 %) | 1 (0.5 %) | 1 (1.7 %) |
| Missing | 86 (78.2%) | 132 (93.0%) | 189 (97.4%) | 136 (98.6%) | 83 (82.2%) | 131 (92.3%) | 186 (96.9%) | 86 (96.6%) | 64 (81.0%) | 127 (97.7%) | 180 (96.8%) | 57 (96.6%) |

| Patient Group | 4 IPF (N=49) | 4 PPF (N=114) | 4 F-ILD (N=154) | 4 F-ILD-U (N=34) | 5 IPF (N=31) | 5 PPF (N=85) | 5 F-ILD (N=114) | 5 F-ILD-U (N=16) |
|---|---|---|---|---|---|---|---|---|
| **Fibrosis status** | | | | | | | | |
| Increased | 8 (16.3 %) | 4 (3.5 %) | 4 (2.6 %) | 0 (0.0 %) | 2 (6.5 %) | 4 (4.7 %) | 4 (3.5 %) | 0 (0.0 %) |
| Decreased | 0 (0.0 %) | 0 (0.0 %) | 1 (0.6 %) | 0 (0.0 %) | 0 (0.0 %) | 0 (0.0 %) | 0 (0.0 %) | 0 (0.0 %) |
| Missing | 41 (83.7%) | 110 (96.5%) | 149 (96.8%) | 34 (100%) | 29 (93.5%) | 81 (95.3%) | 110 (96.5%) | 16 (100%) |

## Lung function during the follow-up years 1-5

| Patient Group | 1 IPF (N=63) | 1 PPF (N=128) | 1 F-ILD (N=134) | 2 IPF (N=52) | 2 PPF (N=104) | 2 F-ILD (N=98) | 3 IPF (N=18) | 3 PPF (N=41) | 3 F-ILD (N=49) |
|---|---|---|---|---|---|---|---|---|---|
| **FVC % predicted** | | | | | | | | | |
| Median (Q1, Q3) | 78.4 (67.2, 88.2) | 71.6 (57.2, 85.9) | 82.5 (69.3, 92.5) | 77.2 (64.5, 87.8) | 72.0 (60.6, 88.3) | 80.3 (72.1, 91.6) | 65.5 (59.9, 82.5) | 69.8 (52.8, 75.3) | 88.1 (77.0, 99.3) |
| Min - Max | 38.9 - 127 | 30.8 - 136 | 36.7 - 157 | 44.0 - 121 | 30.2 - 113 | 43.4 - 131 | 39.0 - 103 | 28.3 - 101 | 46.5 - 149 |

| Patient Group | 4 IPF (N=12) | 4 PPF (N=26) | 4 F-ILD (N=46) | 5 IPF (N=20) | 5 PPF (N=40) | 5 F-ILD (N=46) |
|---|---|---|---|---|---|---|
| **FVC % predicted** | | | | | | |
| Median (Q1, Q3) | 75.3 (50.5, 84.2) | 78.1 (56.1, 96.1) | 81.3 (66.1, 98.2) | 76.3 (62.5, 101) | 77.4 (62.4, 84.1) | 80.5 (69.8, 94.6) |
| Min - Max | 36.4 - 113 | 15.4 - 116 | 40.0 - 139 | 49.3 - 123 | 45.0 - 113 | 38.5 - 150 |

| Patient Group | 1 IPF (N=67) | 1 PPF (N=80) | 1 F-ILD (N=104) | 1 F-ILD-U (N=25) | 2 IPF (N=59) | 2 PPF (N=68) | 2 F-ILD (N=92) | 2 F-ILD-U (N=22) |
|---|---|---|---|---|---|---|---|---|
| **DLCO % predicted** | | | | | | | | |
| Median (Q1, Q3) | 53.8 (42.6, 59.9) | 53.0 (42.6, 62.1) | 63.2 (52.4, 77.6) | 67.6 (50.4, 74.0) | 52.1 (40.5, 60.6) | 52.8 (43.4, 61.8) | 61.6 (50.4, 73.8) | 72.5 (61.4, 78.5) |
| Min - Max | 23.4 - 93.6 | 22.5 - 87.9 | 24.5 - 102 | 32.5 - 82.9 | 25.7 - 96.8 | 25.5 - 103 | 23.8 - 114 | 44.9 - 89.0 |

| Patient Group | 3 IPF (N=34) | 3 PPF (N=55) | 3 F-ILD (N=73) | 3 F-ILD-U (N=5) | 4 IPF (N=23) | 4 PPF (N=36) | 4 F-ILD (N=49) | 4 F-ILD-U (N=2) | 5 IPF (N=15) | 5 PPF (N=25) | 5 F-ILD (N=28) |
|---|---|---|---|---|---|---|---|---|---|---|---|
| **DLCO % predicted** | | | | | | | | | | | |
| Median (Q1, Q3) | 51.2 (42.9, 65.2) | 54.7 (45.2, 68.0) | 61.7 (48.1, 75.3) | 54.5 (50.4, 72.9) | 49.8 (43.0, 61.8) | 56.5 (47.1, 64.2) | 59.1 (51.9, 77.6) | 55.1 (46.1, 64.0) | 54.1 (45.3, 65.2) | 51.7 (41.0, 67.0) | 70.0 (54.6, 82.0) |
| Min - Max | 22.1 - 140 | 13.5 - 99.6 | 20.0 - 106 | 43.1 - 80.3 | 28.2 - 77.3 | 29.3 - 89.6 | 26.1 - 115 | 37.2 - 72.9 | 13.3 - 69.2 | 20.9 - 89.4 | 39.7 - 96.5 |

| Patient Group | 1 IPF (N=67) | 1 PPF (N=80) | 1 F-ILD (N=104) | 1 F-ILD-U (N=25) | 2 IPF (N=59) | 2 PPF (N=68) | 2 F-ILD (N=92) | 2 F-ILD-U (N=22) |
|---|---|---|---|---|---|---|---|---|
| **TLC % predicted** | | | | | | | | |
| Median (Q1, Q3) | 69.5 (62.4, 81.8) | 70.8 (61.0, 83.4) | 78.8 (69.9, 88.8) | 81.0 (73.5, 85.3) | 70.9 (63.8, 79.2) | 71.4 (62.3, 83.8) | 78.5 (68.4, 89.6) | 83.8 (76.1, 86.0) |
| Min - Max | 47.2 - 103 | 41.0 - 101 | 46.2 - 120 | 43.8 - 125 | 50.8 - 110 | 42.1 - 112 | 47.5 - 119 | 65.2 - 99.8 |

| Patient Group | 3 IPF (N=34) | 3 PPF (N=55) | 3 F-ILD (N=73) | 3 F-ILD-U (N=5) | 4 IPF (N=23) | 4 PPF (N=36) | 4 F-ILD (N=49) | 4 F-ILD-U (N=2) | 5 IPF (N=15) | 5 PPF (N=25) | 5 F-ILD (N=28) |
|---|---|---|---|---|---|---|---|---|---|---|---|
| **TLC % predicted** | | | | | | | | | | | |
| Median (Q1, Q3) | 70.5 (61.9, 79.1) | 75.5 (64.0, 84.8) | 82.0 (69.9, 92.3) | 85.4 (75.8, 86.8) | 70.7 (60.3, 78.5) | 72.6 (62.4, 80.6) | 78.6 (66.5, 91.8) | 81.7 (78.7, 84.8) | 71.9 (61.8, 82.5) | 66.4 (55.7, 74.8) | 80.8 (75.0, 90.4) |
| Min - Max | 46.9 - 175 | 36.1 - 162 | 47.9 - 127 | 64.5 - 89.8 | 49.2 - 99.0 | 49.2 - 101 | 47.2 - 119 | 75.6 - 87.8 | 53.6 - 98.1 | 45.0 - 99.3 | 49.4 - 123 |

# BMI during the follow-up years 1-5

| Patient Group | 1 IPF (N=110) | 1 PPF (N=142) | 1 F-ILD (N=194) | 1 F-ILD-U (N=138) | 2 IPF (N=101) | 2 PPF (N=142) | 2 F-ILD (N=192) | 2 F-ILD-U (N=89) |
|---|---|---|---|---|---|---|---|---|
| **Body mass index (BMI)** | | | | | | | | |
| Mean (SD) | 28.6 (5.49) | 28.1 (5.09) | 28.5 (5.29) | 26.4 (5.79) | 27.7 (4.57) | 27.9 (4.99) | 28.1 (4.68) | 27.3 (5.48) |
| Median (Q1, Q3) | 27.2 (25.0, 32.0) | 27.9 (24.3, 31.1) | 28.0 (25.1, 30.8) | 25.7 (22.5, 29.5) | 26.7 (24.5, 30.1) | 27.9 (24.0, 30.5) | 27.5 (25.4, 30.2) | 26.7 (23.4, 30.1) |
| Min - Max | 19.7 - 53.9 | 18.7 - 43.4 | 19.6 - 45.2 | 14.7 - 39.7 | 19.9 - 38.0 | 18.0 - 43.8 | 18.4 - 44.1 | 19.1 - 42.4 |
| Missing | 19 (17.3%) | 10 (7.0%) | 33 (17.0%) | 84 (60.9%) | 25 (24.8%) | 28 (19.7%) | 53 (27.6%) | 47 (52.8%) |

| Patient Group | 3 IPF (N=79) | 3 PPF (N=130) | 3 F-ILD (N=186) | 3 F-ILD-U (N=59) | 4 IPF (N=49) | 4 PPF (N=114) | 4 F-ILD (N=154) | 4 F-ILD-U (N=34) |
|---|---|---|---|---|---|---|---|---|
| **Body mass index (BMI)** | | | | | | | | |
| Mean (SD) | 27.7 (4.49) | 27.7 (5.04) | 28.2 (5.27) | 28.0 (6.40) | 27.4 (4.91) | 27.5 (5.31) | 27.8 (5.56) | 27.5 (6.34) |
| Median (Q1, Q3) | 26.6 (24.5, 29.7) | 27.2 (24.5, 30.4) | 27.8 (24.8, 30.9) | 27.5 (23.9, 32.7) | 26.0 (24.0, 30.5) | 26.6 (23.9, 29.8) | 27.6 (24.5, 30.2) | 26.8 (23.3, 32.0) |
| Min - Max | 20.6 - 40.3 | 16.9 - 43.7 | 18.5 - 41.1 | 15.1 - 44.4 | 17.4 - 37.5 | 18.8 - 48.1 | 15.7 - 44.1 | 15.6 - 42.2 |
| Missing | 25 (31.6%) | 31 (23.8%) | 67 (36.0%) | 37 (62.7%) | 14 (28.6%) | 37 (32.5%) | 50 (32.5%) | 15 (44.1%) |

| Patient Group | 5 IPF (N=31) | 5 PPF (N=85) | 5 F-ILD (N=114) | 5 F-ILD-U (N=16) |
|---|---|---|---|---|
| **Body mass index (BMI)** | | | | |
| Mean (SD) | 27.0 (4.20) | 26.9 (4.21) | 27.8 (5.14) | 28.0 (6.38) |
| Median (Q1, Q3) | 26.3 (24.3, 28.4) | 26.5 (24.3, 29.6) | 27.3 (24.8, 30.2) | 28.4 (25.3, 32.7) |
| Min - Max | 19.4 - 36.8 | 17.2 - 39.6 | 17.8 - 39.5 | 15.2 - 37.1 |
| Missing | 6 (19.4%) | 28 (32.9%) | 36 (31.6%) | 5 (31.3%) |
